# Social Media and the Orthopaedic Surgery Residency Application Process

**DOI:** 10.7759/cureus.23933

**Published:** 2022-04-07

**Authors:** Andrew Butler, Chandler Berke, Usman Zareef, Amr M Tawfik, Bobby Varghese, Giulia Frias, Charles Gatt, Brian M Katt

**Affiliations:** 1 Department of Orthopaedic Surgery, Rutgers Robert Wood Johnson Medical School, New Brunswick, USA

**Keywords:** residency program resources, social media platform, effects of social media, medical education research, orthopaedic residency

## Abstract

Introduction: Social media use in the residency application process has been on the rise, yet applicant and program director (PD) perspectives in orthopaedic residency have not been explored in depth. Our objective is to evaluate perspectives of orthopaedic residency applicants and PDs on social media usage and its impact on the residency application process.

Methods: Cross-sectional survey study conducted in 2021 with two related, anonymous surveys sent to residency applicants and PDs. One hundred thirteen of 562 (20.1%) applicants to our institution and 29 of 148 (19.6%) PDs listed on the Accreditation Council for Graduate Medical Education (ACGME) website responded to our survey and were included in the analysis.

Results: Applicants reported learning about residency programs through Instagram (69.2%), the program website (58.9%), and Doximity (29.9%). Thirty-four percent of applicants reported social media influencing their rank list, with non-training-related posts being the most impactful. PDs reported that 97% of their programs have an official web page, 41% have an active Instagram site, 27% have a Twitter account but none regularly update Doximity. Just over 48% of PDs reported institutional support for online content creation. Financial investment varied, with 35% reporting no spending, and 24% spending over $2501. In response to coronavirus disease 2019 (COVID-19) restrictions, 79% of PDs reported generating increased social media content.

Discussion: Social media provides a low-cost but far-reaching opportunity for PDs to recruit residents and highlight their respective programs. Social media content should display the culture and lifestyle of the program, with consistency in content creation. PDs should also ensure accuracy on external sites such as Doximity.

## Introduction

As it continues to gain popularity, social media will have a role in the residency application process for years to come. The primary aim of this study is to evaluate the current perspectives of applicants and program directors (PD) to better understand and evaluate social media usage and its impacts on the orthopaedic surgery residency application process. 

Orthopaedic surgery residency programs have heavily valued in-person exposure to applicants through away rotations and the interview [1]. These experiences allow for evaluation of applicant "fit," professional potential, and clinical aptitude, which programs historically reference as important qualities in prospective residents [2,3]. Applicants similarly value these experiences as they give insight into resident camaraderie and quality of life [4]. However, the coronavirus disease 2019 (COVID-19) pandemic led to the cancellation of most in-person activities; instead, orthopaedic programs were faced with the task of virtually recreating many aspects of the process [1,5]. One modality that programs utilized as a recruitment tool was social media.

Prior studies have looked at social media use in residency, but limited data exist for orthopaedic surgery specifically, and almost none specifically aimed at its impact on the resident application process [6-8]. Social media use for the residency application process was already on the rise prior to COVID-19; however, orthopaedic surgery departments were slow to follow this trend [6,9-11]. Programs have used social media to showcase their program culture and resident life as well as highlight a wide variety of events from Grand Rounds to resident social gatherings. A recent study reported that social media posts by residency programs influenced 28% of applicants' list of programs to which they applied. The pandemic accelerated the adoption of social media in the residency application process, as it was one of the few exposures applicants had to a program [7].

Additionally, the applicants' view only makes up half of the picture. Little is currently known about the content posted by programs, the cost to the programs, and who is creating and posting on behalf of the program. Orthopaedic Program Directors can help illuminate these factors for their programs and may play a role in managing, advising, and curating on these mediums. An in-depth evaluation of the interplay of social media and the orthopaedic surgery residency process is indicated to inform its future usage in engaging potential orthopaedic applicants.

## Materials and methods

This study was designed as a cross-sectional survey study of applicants and PDs in orthopaedic surgery. Prior to the initiation of this study, institutional review board exemption was acquired per our institution’s protocol. The applicant cohort included fourth-year medical students who applied to our institution’s residency program. All applicant emails were acquired from the Electronic Residency Application Service (ERAS). The PD cohort included all PDs of the Accreditation Council for Graduate Medical Education (ACGME)-accredited orthopaedic surgery residency programs. All PD emails were acquired through the publicly available data on the ACGME website.

**Figure 1:**
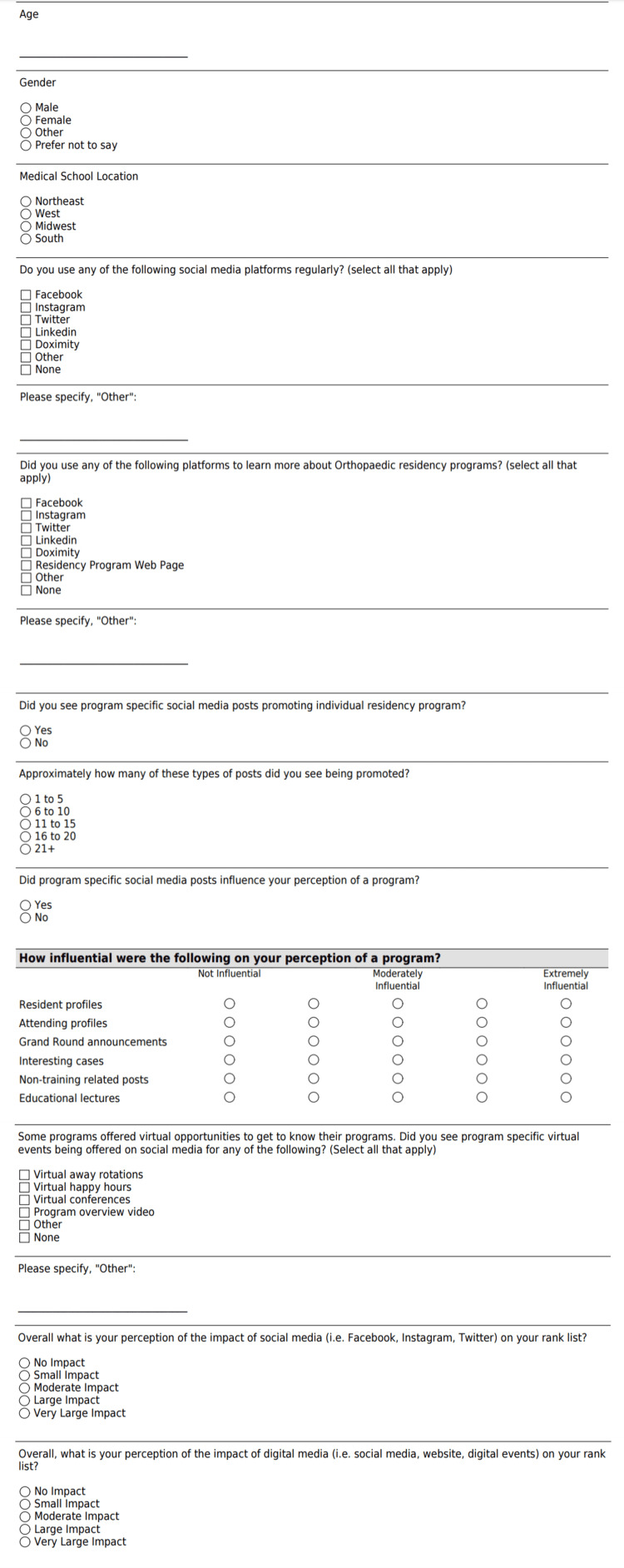
Applicant Survey on Social Media Use

**Figure 2:**
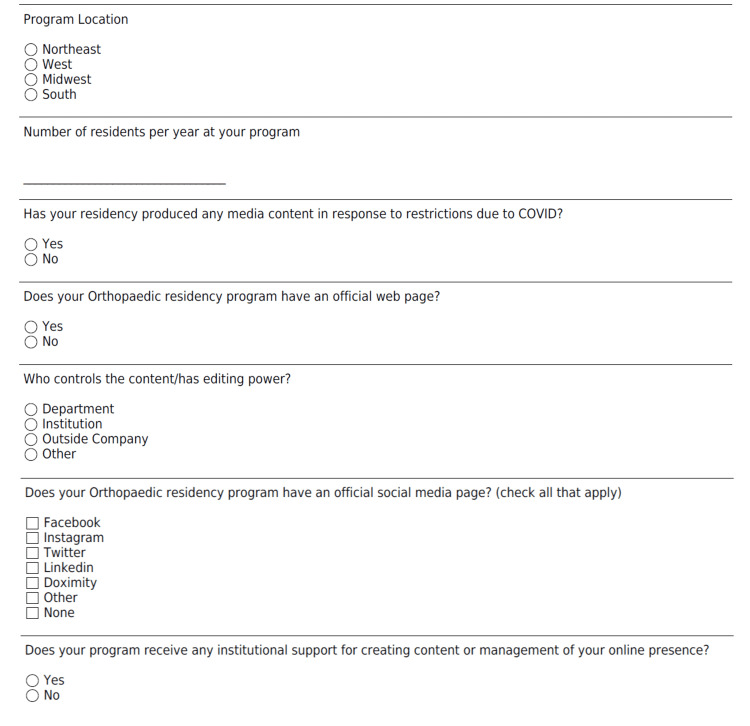
Program Director Survey on Social Media Use

We designed two related anonymous surveys intended to capture the perspectives of applicants and PDs on the use of social media during the application process and its impact on their rank list. The full contents of the applicant and PD surveys can be found in Figure 1 and Figure 2, respectively.

Between February 21st and March 15th 2021, the anonymous surveys were distributed to our acquired email lists. Applicants who completed the survey were offered a chance to win one of 10 $50 Amazon gift cards. Email reminders were sent to applicants and PDs who had not yet completed the survey for a total of three cycles scheduled five days apart. One final email was sent to both applicants and PDs on March 13th with a modification to ask for their assistance in adequately powering the study.

Survey responses were collected and analyzed using R Statistical Software (R Core Team, 2017). Continuous data were reported as mean and standard deviation, with categorical data reported as a percentage of the total group. Categorical Likert-style response data were assigned numerical values (from 1-5) and were reported as weighted averages.

## Results

The survey was successfully sent to a total of 562 applicants and 148 PD emails. There were a total of 1289 orthopedic applicants in 2020 [12]. One hundred thirteen applicants completed the survey (20.1% response rate). Twenty-nine PDs completed the survey (19.6% response rate).

Applicants

Applicants who responded to the survey were 27.9 ± 3.0 years old, with 69.1% identifying as male and 30.0% identifying as female. The geographic distribution of applicant responses was 53.6% Northeast, 20.9% South, 20.0% Midwest, and 5.5% West.

Table 1 summarizes applicant perceptions of social media use. The most commonly used social media platforms by applicants in their public lives were Instagram (71.7%), Facebook (53.8%), and Twitter (33.0%). Regarding learning about orthopaedic residency programs, applicants most used Instagram (69.2%), the residency program website (58.9%), and Doximity (29.9%).

**Table 1:** Orthopaedic Applicant Use of Social Media for the Residency Application Process *Respondents reported the data for this section categorically (from no impact = 1 to very large impact = 5). These responses were converted to numerical scores and presented as weighted averages.

Do you use any of the following social media platforms regularly?	
	Instagram	71.7%
Facebook	53.8%
Twitter	33.0%
Doximity	18.9%
LinkedIn	15.1%
None	15.1%
Other	2.8%
Did you use any of the following platforms to learn more about Orthopaedic residency programs?	
	Instagram	69.2%
	Residency Program Website	58.9%
Doximity	29.9%
Twitter	24.3%
Facebook	6.5%
LinkedIn	2.8%
None	10.3%
Other	4.7%
Did you see program specific social media posts promoting individual residency programs?	
	Yes	69.2%
	No	30.8%
Approximately how many of these types of posts did you see being promoted?	
	1 to 5	16.2%
6 to 10	17.6%
11 to 15	16.2%
16 to 20	4.1%
21+	45.9%
Did program specific social media posts influence your perception of a program?	
	Yes	38.2%
No	61.8%
How influential were the following on your perception of a program?*	
	Resident profiles	3.24 ± 1.04
Attending profiles	2.59 ± 1.12
Grand Round announcements	1.90 ± 0.97
Interesting Cases	2.54 ± 1.00
Non-training related posts	3.76 ± 0.99
Educational lectures	2.49 ± 0.93
Some programs offered virtual opportunities to get to know their programs. Did you see program specific virtual events being offered on social media for any of the following?	
Virtual happy hours	59.3%
Program overview video	51.6%
Virtual conferences	38.5%
Virtual away rotations	26.4%
None	28.6%
Other	0.0%
Overall, what is your perception of the impact of social media?*	2.20 ± 0.96
Overall, what is your perception of the impact of digital media?*	2.44 ± 1.17

Respondents reported that social media had a large or very large impact on the rank list for 8.8%, a moderate impact for 24.2%, and a small or no impact for 67.1% of applicants. When all digital media was considered, there was a reported large or very large impact on the rank list for 18.7%, a moderate impact for 29.7%, and a small or no impact for 51.7% of applicants.

Of those who indicated they were influenced by the social media posts, non-training related posts and resident profiles had the most impact on their rank list. Many applicants reported seeing virtual opportunities being advertised by programs on social media. The opportunities that applicants saw offered most were virtual happy hours (59.3%), an overview video for the program (51.6%), and virtual conferences (38.5%).

Table 2 summarizes applicant reported influence by social media. Just over 69% (N=74) reported that they saw social media posts promoting an individual residency program. Of those who saw posts, 69% (N=40) reported being influenced by the posts. Those who had not seen social media posts were removed from subsequent questions regarding the frequency of social media posts. Those who reported being influenced were more likely to report seeing social media posts (p < 0.001). Additionally, those who saw more posts were more likely to indicate they were influenced by social media (p = 0.024).

**Table 2:** Applicant Reported Influence of Social Media Posts on Their Opinion of Residency Programs Based on Social Media Posts Seen Nonparametric continuous data is presented as median [1st quartile, 3rd quartile]. Categorical data is presented as count (% of total count). P-values for continuous data were calculated by performing Mann Whitney tests. Chi-Square tests were used to calculate p-values for categorical data.

	Influenced	Not Influenced	P-value
	(n = 42)	(n = 68)	
Age	27.0 [26.0-28.0]	28.0 [26.0-29.2]	0.123
Sex			0.106
Male	25 (59.5)	51 (75.0)	
Female	17 (40.5)	16 (23.5)	
Prefer Not to Say	0 (0.00)	1 (1.47)	
Did You See Social Media Posts			<0.001
Yes	40 (97.6)	34 (51.5)	
No	1 (2.44)	32 (48.5)	
Number of Posts Seen			0.024
1 to 5	4 (10.0)	8 (23.5)	
6 to 10	3 (7.50)	10 (29.4)	
11 to 15	8 (20.0)	4 (11.8)	
16 to 20	2 (5.00)	1 (2.94)	
21+	23 (57.5)	11 (32.4)	

Program directors

The geographic distribution of PD responses was 31.0% Northeast, 31.0% Midwest, 24.1% South, and 13.8% West. The average number of residents per year at these programs was 4.8 ± 3.3.

The majority of orthopaedic residency programs (96.6%) reported having an official web page. For 64.3%, the department controls the content and has editing power. For the remaining 35.7%, the institution has control. The most used social media pages for residency programs were Instagram (41.4%), Twitter (27.6%), and Facebook (20.7%). However, 41.4% of programs reported having no official social media page. The social media content on these pages is most frequently managed by a resident (50.0%), program director (34.6%), or program administrator/coordinator (30.8%). Less than half (48.3%) of programs receive institutional support for online content creation. Social media spending was highly varied, with 34.5% of PDs reporting no spending, 24.1% of programs reported spending over $2501, and 3.4% (one program) spending >$10,000. Despite this, 71.4% of programs report that this amount was adequate to meet their needs (Table 3).

**Table 3:** Program Director Use of Social Media for the Residency Application Process

Does your Orthopaedic residency program have an official web page?
Yes	96.60%	
No	3.40%	
Who controls the content/has editing power?		
Department	64.30%	
Institution	35.70%	
Outside Company	0.00%	
Other	0.00%	
Does your Orthopaedic residency program have an official social media page?	
Instagram	41.4%	
Twitter	27.6%	
Facebook	20.7%	
LinkedIn	3.4%	
Doximity	0.0%	
None	41.4%	
Other	3.4%	
Who manages/posts on your program's social media page?		
Resident	50.0%	
Program Director	34.6%	
Program Administrator/Coordinator	30.8%	
Faculty/Attending	19.2%	
Professional or Company outside of the department	0.0%	
Other	23.1%	
Has your residency produced any media content in response to restrictions due to COVID?		
Yes	79.3%	
No	20.7%	
What types of specific social media posts did your program produce?		
Resident profiles	48.0%	
Non-training related posts	44.0%	
Attending profiles	28.0%	
Grand Round announcements	20.0%	
Interesting Cases	12.0%	
Other	32.0%	
Does your program receive any institutional support for creating content or management of your online presence?		
Yes	48.3%	
No	51.7%	
What was your approximate total amount spent on social media/virtual recruitment for the year?		
$0	34.5%	
$1-1000	37.9%	
$1001-2500	3.4%	
$2501-5000	13.8%	
$5001-7500	6.9%	
$7501-10000	0.0%	
$10001+	3.4%	
Do you think that the amount spent was adequate to meet your needs?		
Yes	71.4%	
No	28.6%	

In response to COVID restrictions, 79.3% of programs report producing additional social media content. This content included resident profiles (48.0%), non-training related posts (44.0%), and attending profiles (28.0%).

## Discussion

Motivated by the realities of recruitment during COVID-19, social media usage is rising in usage for the residency application process by both residency programs and applicants. This rise in usage parallels results from Economides et al. that showed the views on social media use among applicants and trainees has shifted towards utilizing its potential for professional and scholastic development [13].

In our study, applicants found non-training-related posts, such as departmental social outings and resident profiles, to be the most influential for their perception of a program. These findings are consistent with previous research where Huntington et al. cited perceived resident happiness and camaraderie as two of the most important factors when creating an applicant rank list [10]. Additionally, the 2019 National Residency Match Program (NRMP) applicant survey found the same two factors to be among the most important to residents when creating their rank lists [14]. Despite this, less than half of PDs in this study reported posting resident profiles and non-training-related content on social media. Additionally, 69% of applicants reported using Instagram specifically to find out about residency programs, yet only 41% of programs reported having a program Instagram account. Our study highlights these discrepancies between applicant preferences and program offerings. Social media presents a unique opportunity to showcase the program's culture and attract prospective applicants. Narrowing the discrepancy between a program's social media content and an applicant's preferences is an opportunity to tailor program content to meet the intended audience's needs and expectations. This also presents an area of future study to evaluate the optimum strategy, including the number of posts per week and content of posts to increase visibility, influence, and brand.

Despite the widespread usage of social media by orthopaedic surgery applicants, we found respondents did not report that these platforms play a significant role in influencing their rank lists. This is consistent with the current state of the literature in orthopaedic surgery, where traditional variables such as program reputation, interview experience, and geographic location are still listed as the most influential factors in applicant rank lists [3,7]. While not being reported as having a significant influence on rank lists, our study did show that those who saw more posts were more likely to report that social media posts were influential compared to those who did not see any or only saw a few posts. While our study was not explicitly designed to find the optimum number of posts, it is clear that those who viewed over 21 posts were more likely to say they were influenced. This is in line with current literature outside of medicine, where studies have shown the power and influence of social media posts on beliefs and perceptions [15]. Research in other fields has shown that media is influential regardless of perceived impact [15,16]. Therefore, social media remains a strong opportunity to influence brand perception based on applicants' reported usage of social media platforms. Additional research should be conducted to better identify strategies and factors that affect and promote a program's brand and perceived prestige.

While the COVID-19 pandemic was a pivotal component in forcing the recent system-wide reliance on social media, data from previous years showed an upward trend in social media usage [8,9]. While in-person experiences will return, programs should continue to utilize social media as a low-cost but far-reaching recruitment approach. The majority of programs spent under $1000 during the 2020-2021 application cycle; however, 24.1% of programs reported spending $2500 or more. Further exploration is needed to better understand the higher cost content utilized by some programs in 2020-2021. Incorporating social media to bolster recruitment is a relatively low-cost investment; our results suggest an opportunity to explore a cost-benefit analysis of various virtual recruitment content to increase visibility and reach.

In our study, almost one-third of applicants used Doximity to learn about programs; however, no PDs reported Doximity usage. While this is considerably lower than what is reported in applicants outside of othopaedics, current literature shows Doximity's influence on applicants' program applications, interview acceptance, and match list ranking decisions [17,18]. Because of Doximity's potential usage in resident recruitment, we recommend PDs view and if needed modify their Doximity profiles to ensure accuracy and collaborate with Doximity to rectify this information.

We are aware of a few limitations with our study. Due to the study's cross-sectional design, it is unclear if the associations identified in this study were causal. The survey was only sent to orthopaedic applicants at a single institution; however, this cohort represented approximately half of the year's applicant pool. Additionally, the survey response rate was approximately 20%, limiting the ability to generalize our findings; however, this is consistent with response rates found in the literature [19]. Finally, since our survey was focused on digital and social media use, it is possible that only those applicants and PDs who utilize these applications responded to the study. This may have potentially inflated our estimates of social media use among applicants and orthopaedic residency programs.

The increasing use of social media as a potential recruitment tool was accelerated by COVID-19 and provides a promising opportunity for orthopaedic programs to refine their brand and extend their reach. Our study highlights the attitudes of applicants towards particular social media platforms. PDs can modify their social media approach to tailor content towards applicant preference.

## Conclusions

Programs should continue to utilize social media as a low-cost but far-reaching recruitment approach to augment their in-person experiences. Social media provides a unique and challenging opportunity to cultivate a program's brand, which requires attention and resources to maintain. Applicants want to know about the culture of the program. Programs should use social media platforms to highlight culture and lifestyle, factors that are difficult to showcase in other interactions. Programs should aim for a minimum of two posts per month to increase the likelihood of posts being seen and influencing applicants.
